# Physiological responses and adaptive mechanisms of amphibians and reptiles to multiple interacting environmental stressors: an integrative review

**DOI:** 10.3389/fphys.2026.1785483

**Published:** 2026-04-30

**Authors:** Muammer Kurnaz

**Affiliations:** Department of Medical Services and Techniques, Kelkit Sema Doğan Vocational School of Helth Services, Gümüşhane University, Gümüşhane, Türkiye

**Keywords:** climate change, conservation physiology, ectotherms, herpetofaunal decline, infectious disease, osmoregulation, physiological plasticity, reptile ecophysiology

## Abstract

This integrative review synthesizes current knowledge on the physiological responses and adaptive mechanisms of amphibians and reptiles to multiple interacting environmental stressors, with particular emphasis on synergistic effects among temperature, hydric stress, disease, and pollution. Given the stronger empirical basis for amphibians in the existing literature, amphibian responses are covered in greater depth, while reptile-specific physiology, immunology, and emerging infectious diseases are explicitly addressed in dedicated sections throughout the review. Critical thermal tolerance analyses reveal that approximately 7.5% of amphibian species will exceed their physiological limits under a 4 °C warming scenario, with tropical lowland species already operating near their CTmax thresholds. Thermal plasticity is limited, with acclimation responses averaging only 0.13 °C increase in CTmax per 1 °C environmental warming—insufficient to track rapid climate change. Water balance regulation shows dramatic interspecific variation, with cutaneous resistance ranging from 0.05 s/cm in aquatic amphibians to >1000 s/cm in desert-adapted reptiles. Synergistic interactions between thermal and hydric stress significantly amplify vulnerability, particularly in dehydration scenarios that reduce critical thermal limits. Chemical pollutants, including heavy metals and pesticides, cause developmental abnormalities (535% increase in malformation frequency), immunosuppression, and endocrine disruption across multiple life stages. Emerging infectious diseases, particularly chytridiomycosis (*Batrachochytrium dendrobatidis* and *B. salamandrivorans*) and ranaviruses, drive mass mortality events globally, with co-infections exacerbating population declines. Climate change intensifies disease susceptibility through stress-mediated immunosuppression and altered pathogen dynamics. Adaptive capacity varies markedly among species. While amphibians exhibit strong phenological responses (2-4× greater than other taxa), genetic adaptation potential remains limited by narrow dispersal abilities and habitat fragmentation. Microhabitat buffering can reduce thermal extremes by several degrees but depends critically on habitat structural integrity. This review demonstrates that the pace of anthropogenic change challenges the adaptive capacity of most species, necessitating integrated conservation strategies including microhabitat preservation, climate corridor establishment, pollution mitigation, disease surveillance, and *ex-situ* conservation programs.

## Introduction

Amphibians (Amphibia) and reptiles (Reptilia) are ectotherm vertebrate groups that play critical roles in the structural and functional integrity of global biodiversity. Because their energy budgets, physiological performance, and life cycle processes are largely dependent on ambient temperature, these taxa are particularly sensitive to environmental changes (Nowakowski et al., 2018; Cox et al., 2022; Hayden Bofill and Blom, 2024). Since metabolic rate, immune responses, growth, and reproductive success in ectothermic organisms are directly determined by environmental temperature regimes, climatic fluctuations and habitat changes have decisive effects on population dynamics (Huey et al., 2012; Deutsch et al., 2008). Previous reviews have examined individual stressors such as climate warming, pollution, and emerging diseases in amphibians and reptiles (Blaustein et al., 2011; Hayes et al., 2010; Urban et al., 2013; Cox et al., 2022). However, fewer studies have integrated the physiological consequences of multiple interacting stressors. The present review addresses this gap by synthesizing mechanistic evidence across thermal, hydric, chemical, and pathogenic stressors within a unified physiological framework.

Over the past few decades, habitat loss and fragmentation, global climate change, chemical pollution, and newly emerging pathogens have created simultaneous and often synergistic pressures on amphibian and reptile populations (Gibbons et al., 2000; Houlahan et al., 2000; Wake and Vredenburg, 2008). In particular, rapid land-use changes and shifts in temperature and humidity regimes are pushing the thermal niche limits of these groups and leading to stress conditions that exceed their adaptive capacity (Sinervo et al., 2010; Urban, 2015).

Throughout this review, several technical terms are used repeatedly and are defined here for clarity. Critical thermal maximum (CTmax) refers to the upper body temperature at which an organism loses coordinated locomotion and becomes unable to escape lethal conditions (Lutterschmidt and Hutchison, 1997); critical thermal minimum (CTmin) is the analogous lower temperature threshold. The thermal safety margin (TSM) quantifies the difference between an organism’s CTmax and the maximum environmental temperature it normally experiences, and provides an index of vulnerability to warming (Deutsch et al., 2008). Ectothermy describes the dependence on external environmental heat sources for body temperature regulation, in contrast to endothermy. The term stressor denotes any environmental factor that imposes a physiological demand exceeding the organism’s homeostatic capacity; synergistic effects occur when two or more stressors interact to produce a combined impact greater than the sum of their individual effects. Chytridiomycosis refers to the infectious disease caused by chytrid fungi of the genus Batrachochytrium (Bd: B. dendrobatidis; Bsal: B. salamandrivorans), responsible for global amphibian declines (Berger et al., 1998). Hydroregulation denotes the suite of behavioral, morphological, and physiological mechanisms by which organisms regulate body water content. These definitions provide the conceptual foundation for the integrative synthesis that follows.

Amphibians are currently identified as the group with the highest extinction and population decline rates globally among vertebrates (Blaustein and Kiesecker, 2002; Stuart et al., 2004). Highly permeable skin structures, complex life cycles encompassing both aquatic and terrestrial phases, and generally limited dispersal capabilities make amphibians extremely vulnerable to environmental stressors (Kerby et al., 2010; Wells, 2007). These characteristics also make amphibians early warning indicators (bioindicators) of environmental degradation (Blaustein et al., 2011).

Reptiles, on the other hand, have been able to adapt to drier and more variable habitats by significantly reducing their dependence on water thanks to their keratinized epidermis and amniotic egg adaptations (Pough, 1983; Packard and Packard, 1988). However, recent global assessments have revealed that reptiles are also more threatened than expected and exhibit significant sensitivity to climate change and habitat loss (Cox et al., 2022; Böhm et al., 2013). Narrow thermal tolerance limits and excessive reliance on behavioral thermoregulation put many reptile species at risk in the face of rapidly changing environmental conditions (Kearney et al., 2009; Clusella-Trullas and Chown, 2014).

Although both taxa have developed adaptive mechanisms such as physiological plasticity, behavioral thermoregulation, and microhabitat selection in response to environmental variability, the speed and severity of current environmental changes limit the effectiveness of these adaptations (Seebacher et al., 2015; Gunderson and Stillman, 2015). In this context, a holistic assessment of the responses of amphibians and reptiles to global environmental changes is of great importance both for understanding fundamental ecological processes and for developing effective conservation strategies.

The main objective of this review is to synthesize the physiological responses and adaptive mechanisms of amphibians and reptiles under multiple interacting environmental stressors. The review integrates mechanistic evidence across thermal, hydric, chemical, and pathogenic pressures, with emphasis on synergistic effects that amplify vulnerability beyond single-stressor impacts. While the existing scientific literature provides a substantially richer empirical basis for amphibians — particularly in disease physiology, immunotoxicology, and chytrid biology — reptile-specific physiology, immunology, and emerging infectious diseases (Snake Fungal Disease, chelonian herpesvirus, ranavirus cross-infections) are explicitly addressed in dedicated subsections. This review therefore does not treat the two groups as equivalent in terms of literature depth, but rather maps the state of knowledge for each where robust evidence exists, and identifies critical knowledge gaps for reptile ecophysiology. The taxonomic asymmetry in the literature reflects genuine data limitations rather than editorial choice, and is itself an important finding communicated to the reader.

## Literature search strategy

This review study synthesized peer-reviewed literature published primarily between 2000 and 2025, supplemented with earlier foundational studies where necessary to evaluate physiological responses and adaptive mechanisms of amphibians and reptiles to environmental stressors. Comprehensive searches were conducted across multiple scientific databases including Web of Science (Core Collection), PubMed/MEDLINE, Scopus, and Google Scholar (for grey literature and recent preprints).

Search strings combined taxonomic, physiological, and environmental terms using Boolean operators. Core search terms included: (“amphibian*” OR “anuran*” OR “salamander*” OR “reptile*” OR “herpetofauna”) AND (“thermal tolerance” OR “CTmax” OR “critical thermal” OR “climate change” OR “global warming” OR “physiological adaptation” OR “osmoregulation” OR “water balance” OR “chytridiomycosis” OR “Batrachochytrium” OR “ranavirus” OR “pollution” OR “pesticide*” OR “heavy metal*”).

Studies were included if they: (1) focused on amphibian and/or reptile physiological responses, (2) addressed environmental stressors (thermal, hydric, chemical, pathogenic), (3) provided empirical data or mechanistic insights, (4) were published in peer-reviewed journals or established preprint servers, and (5) were written in English. Studies were excluded if they lacked physiological or mechanistic focus, were purely observational without physiological measurements, focused exclusively on non-herpetofaunal taxa, or were inaccessible after institutional access attempts.

A total of 169 primary sources were identified and included in this review. Key findings were extracted, synthesized, and organized thematically according to major stressor categories (thermal, hydric, chemical, disease, climate). Emphasis was placed on: (1) quantitative physiological limits (CTmax, CTmin, cutaneous resistance), (2) mechanistic pathways (osmoregulation, detoxification, immunity), (3) multi-stressor interactions and synergistic effects, and (4) conservation implications and adaptation potential. This integrative approach ensures comprehensive coverage of current knowledge on amphibian and reptile ecophysiology under environmental change.

This study is classified as an integrative review, following the framework proposed by Torraco (2005) and Whittemore and Knafl (2005), which synthesizes diverse theoretical and empirical literature to provide a comprehensive understanding of a phenomenon. Unlike systematic reviews, integrative reviews permit the inclusion of varied methodological designs and do not require exhaustive meta-analytical procedures; however, they demand rigorous and transparent literature search and selection processes. This approach was selected because the existing evidence base on herpetofaunal responses to multiple environmental stressors spans experimental, observational, physiological, and ecological paradigms that cannot be adequately captured by a single-methodology systematic review. The literature screening process followed the general principles of transparent evidence synthesis (Liberati et al., 2009). The initial database search returned approximately 8,500 records, which were screened in three sequential stages: (Stage 1) title and keyword screening excluded approximately 6,200 records clearly outside the scope of amphibian or reptile physiology; (Stage 2) abstract screening excluded approximately 2,000 additional records lacking physiological measurements or mechanistic focus; and (Stage 3) full-text evaluation of the remaining approximately 300 records against all stated inclusion and exclusion criteria yielded a final corpus of 169 sources. Duplicate records identified across databases were removed prior to screening. Discrepancies during full-text evaluation were resolved by iterative review against the inclusion criteria. Geographic and taxonomic biases inherent in this dataset are explicitly acknowledged: the majority of included studies originate from North America, Europe, and South America, reflecting existing publication patterns. Amphibians are substantially better represented than reptiles across all stressor categories, particularly in disease physiology, immunotoxicology, and chytrid biology; this disparity is discussed in the relevant sections below.

### Quality assessment and risk of bias

Given the integrative nature of this review, formal risk-of-bias assessment tools designed for clinical research (e.g., Cochrane RoB 2, Newcastle–Ottawa Scale) were not directly applicable. Instead, a structured methodological quality appraisal was applied to all 169 included primary sources using four evaluative criteria adapted from frameworks recommended for ecological and physiological integrative reviews (Nakagawa and Poulin, 2012; Gurevitch et al., 2018).

Each included study was evaluated against the following criteria: (1) Physiological measurement validity — whether the study employed standardized, replicable methods for quantifying physiological endpoints (e.g., CTmax via critical thermal methodology with reported ramping rate; cutaneous resistance via gravimetric water loss at controlled temperature and humidity); (2) Sample size adequacy — whether the study reported sufficient biological replication to support the reported conclusions (minimum threshold: n ≥ 4 per treatment or taxon group, consistent with the lower bound of reported values across included studies); (3) Experimental or observational rigor — whether confounding variables (e.g., body size, acclimation temperature, season, hydration state) were controlled or statistically accounted for; and (4) Taxonomic and geographic representativeness — whether the study acknowledged the scope limitations of its taxon and geographic sampling.

Studies meeting all four criteria were classified as high quality and given primary evidential weight in the synthesis. Studies meeting three criteria were classified as moderate quality and cited with appropriate caveats. Studies meeting fewer than three criteria were retained only where they represented the sole available evidence for a specific taxon, stressor, or geographic region; in such cases their limitations are explicitly noted in the relevant section. Across the 169 included sources, approximately 61% were classified as high quality, 32% as moderate quality, and 7% as limited quality. The limited-quality studies were concentrated in reptile-specific disease physiology and comparative toxicology — precisely the areas identified as underrepresented in the primary literature, and where knowledge gaps are most acute.

Publication bias was assessed qualitatively by examining the directional distribution of reported effects. The dataset shows a predominance of studies reporting negative physiological consequences of environmental stressors, which may partly reflect publication bias toward significant or alarming findings. Where possible, null or mixed-result studies were actively sought and included to provide a balanced synthesis. Additionally, the geographic and taxonomic biases documented above (North American, European, and South American populations; amphibian-dominated datasets) represent a systematic evidence gap rather than a publication bias per se, and are acknowledged throughout the relevant sections of this review.

## Thermal tolerance and temperature adaptations

### Critical thermal limits and tolerance ranges

As ectothermic vertebrates, amphibians and reptiles depend on environmental temperature to regulate body temperature and physiological performance. The critical thermal maximum (CTmax) and critical thermal minimum (CTmin) define the absolute temperature limits beyond which individuals lose motor coordination, experience metabolic failure, and ultimately die (Lutterschmidt and Hutchison, 1997; von May et al., 2019). These limits vary considerably among species in relation to habitat type, geographic distribution, microclimate conditions, and ontogenetic stage; larval and juvenile stages typically exhibit narrower thermal tolerance windows than adults, constraining population renewal dynamics (Nowakowski et al., 2018; Ruthsatz et al., 2018; Fan et al., 2021; Pottier et al., 2022).

Recent analyses reveal that amphibian species in tropical regions are already operating near their thermal tolerance limits. von May et al. (2019) measured critical thermal limits in 56 lowland Amazonian frog species (representing ~65% of local alpha diversity) and project that approximately 4% will exceed CTmax under a 3 °C warming scenario, while a further 25% will experience moderate physiological stress; these estimates are specific to tropical lowland Amazonian assemblages and cannot be extrapolated globally without caution. At the global scale, Pottier et al. (2025) applied a phylogenetically informed imputation framework to 5,203 amphibian species and found that 2% are currently exposed to overheating events. Despite accounting for heat-tolerance plasticity, a 4 °C global temperature increase would push 7.5% of amphibian species beyond their physiological limits, representing a marked step change in extinction risk (Pottier et al., 2025).

These findings particularly highlight the importance of the concept of thermal safety margin (TSM). TSM refers to the difference between an organism’s CTmax value and the maximum environmental temperature it is exposed to, and is considered a strong indicator of vulnerability to climate change (Deutsch et al., 2008; Huey et al., 2012). It has been reported that these margins are extremely narrow in tropical amphibians and many reptile species, meaning that even small temperature increases can have serious physiological and demographic consequences (Sunday et al., 2014; Sinervo et al., 2010).

While behavioral thermoregulation (e.g., shading, sunbathing, or microhabitat selection) in reptiles can mitigate the effects of thermal stress in the short term, habitat degradation and increased temperature extremes limit the effectiveness of these strategies (Kearney et al., 2009). Therefore, it is becoming increasingly clear that physiological limits such as CTmax and CTmin are key factors determining not only individual tolerance but also the geographical distribution of species and their future extinction risks (**Table 1**).

**Table 1:** Critical thermal limits across major amphibian and reptile groups.

Taxon/species	Habitat type	CTmax (°C)	CTmin (°C)	TSM* (°C)	Reference
Amphibians					
Tropical lowland anurans	Rainforest	35.1 ± 1.5	12.8 ± 2.1	2.1	von May et al., 2019
Temperate anurans	Forest/wetland	34-36	0.5-3.5	8-10	Ruthsatz et al., 2022
Plethodontid salamanders	Forest floor	30.8 ± 1.3	1.5 ± 0.6	5.8	Caruso et al., 2014
Arboreal hylids	Tree canopy	38.2 ± 1.1	8.5 ± 1.2	6.2	Pottier et al., 2022
Reptiles					
Desert lizards	Arid	44-45	5-7	14-15	Sinervo et al., 2010, 2024
Temperate lizards	Terrestrial	40-43	3-5	11-13	Cox et al., 2022
Desert tortoises	Arid	43.2 ± 1.0	7.8 ± 1.2	13.2	Peterson, 1996
Crocodilians	Semi-aquatic	38.8 ± 1.3	10.5 ± 1.8	8.8	Seebacher et al., 1999

*TSM, Thermal Safety Margin (difference between CTmax and maximum habitat temperature). Values represent means ± SD where available; sample sizes range from n = 8 to n = 312 individuals per taxon group across cited studies. CTmax was primarily determined using the critical thermal methodology (ramping rate: 0.25–1.0 °C min⁻¹); inter-study comparability should be interpreted with caution due to methodological heterogeneity. Geographic bias toward Neotropical, North American and European populations is acknowledged; African, Asian, and Australasian taxa are underrepresented. Substantial variation exists among clades, populations, and life stages beyond the ranges shown.

### Thermoregulation mechanisms

Amphibians and reptiles, being ectothermic organisms, regulate their body temperature largely in response to environmental conditions; they employ behavioral thermoregulation mechanisms to maintain optimal physiological performance. These mechanisms allow individuals to maintain their body temperature within a preferred temperature range (Tset), directly impacting growth, digestion, immune function, and reproductive success (Huey, 1982; Angilletta, 2009).

Behavioral thermoregulation strategies generally include the following key components (Huey, 1982; Angilletta, 2009). Heliothermic behavior, increasing body temperature through direct radiant heat uptake via sunbathing. This strategy is common in many reptile species, especially those living in open habitats (Huey and Slatkin, 1976; Seebacher and Franklin, 2005). Tigmothermic behavior, conductive heat gain through contact with warmed rock, soil, or other surfaces. This behavior is particularly important in conditions where ambient temperatures are low, especially at night or in the early morning (Cowles and Bogert, 2006). Microhabitat selection, utilizing thermally more stable microhabitats such as shaded areas, under vegetation, near water sources, or underground shelters. Microhabitat heterogeneity acts as a critical buffer in ectotherms avoiding thermal stress (Kearney et al., 2009). Adjusting activity timing, avoiding periods of extreme heat or cold by varying activity patterns on a daily (diurnal–nocturnal) or seasonal scale. This strategy is particularly evident in species living in hot and arid ecosystems (Adolph and Porter, 1993).

Observations on lizard species living in desert ecosystems reveal that activity timing plays a central role in thermoregulation. Individuals in these species are generally active around midday in spring and autumn, while in summer they shift their activity to early morning and late afternoon hours to avoid extreme heat (Arizona-Sonora Desert Museum). Similar patterns have been supported by experimental and observational studies conducted in different geographies, demonstrating that activity timing is an effective behavioral adaptation that prevents exceeding thermal tolerance limits (Sinervo et al., 2010; Gunderson and Leal, 2015).

However, the effectiveness of behavioral thermoregulation largely depends on habitat structure and microclimate diversity. Habitat loss, vegetation reduction, and land-use changes restrict the accessibility of thermal refuges, reducing the adaptive value of these behavioral strategies (Scheffers et al., 2014; Sunday et al., 2014). Therefore, while behavioral thermoregulation mechanisms can mitigate the effects of thermal stress in the short term, the increasing pace of environmental change limits the long-term protective effect of these strategies.

### Thermal plasticity and acclimation

It has long been known that the thermal tolerance limits of amphibians and reptiles are not fixed, but rather exhibit a certain degree of phenotypic plasticity. Phenotypic plasticity refers to the capacity of individuals to respond physiologically or morphologically to changes in environmental conditions without altering their genetic structure, providing an important buffering mechanism against short-term environmental changes (West-Eberhard, 2003; Angilletta, 2009). In this context, acclimatization processes represent reversible changes in the thermal tolerance limits of organisms when exposed to different temperature regimes.

Extensive acclimatization experiments and comparative analyses show that CTmax values in amphibians and reptiles increase by an average of 0.13 °C for every 1 °C increase in acclimatization temperature (Clusella-Trullas and Chown, 2014). This finding reveals that thermal tolerance in ectothermic vertebrates is flexible to a certain extent, but also shows that this increase is generally limited compared to the rate of environmental temperature changes. Indeed, a meta-analysis by Seebacher et al. (2015) revealed that amphibians and reptiles have limited acclimatization capacity, particularly in subcritical temperature ranges, increasing their vulnerability to extreme heat events.

While it has been suggested that phenotypic plasticity can enhance species resilience to climate change, the effectiveness of this mechanism varies significantly across species, populations, and life stages (Gunderson and Stillman, 2015). Urban et al. (2016) emphasized the need to consider phenotypic plasticity and genetic adaptation together in amphibians and reptiles, suggesting that these two mechanisms could potentially mitigate the negative impacts of climate change. However, the evolutionary timescales required for the emergence of adaptive responses often do not coincide with the pace of rapid environmental changes observed today (Radchuk et al., 2019).

Long-term field studies demonstrate that adaptive responses to thermal changes encompass not only physiological but also morphological and life history characteristics. A 55-year monitoring study of plethodontid salamander populations in the Appalachian Mountains revealed significant reductions in body size in six species, suggesting that this reduction may be a potential adaptive response to increasing environmental temperatures (Caruso et al., 2014). Similar size reduction trends have been reported in different ectothermic groups, suggesting that this phenomenon may be a strategy to reduce energy balance and water loss under thermal stress (Dillon et al., 2010; Gardner et al., 2011; Sheridan and Bickford, 2011). Although smaller body size increases the surface-area-to-volume ratio, it may also reduce absolute water demand, shorten development time, and lower total metabolic requirements, potentially providing an energetic advantage under warming conditions.

### Relationship between metabolism and temperature

In ectothermic vertebrates, metabolic rate is tightly coupled to body temperature through the temperature-dependent kinetics of enzymatic reactions. As formalized by Gillooly et al. (2001), metabolic rate increases exponentially with temperature; even modest environmental warming therefore elevates oxygen demand, energetic expenditure, and water loss. Because amphibians and reptiles do not generate metabolic heat as endotherms do, this coupling is largely passive and direct (Halsey et al., 2018).

Since the regulation of body temperature in amphibians and reptiles does not involve a mechanism based on metabolic heat production as in mammals and birds, the effect of temperature on metabolism is largely a result of passive physiological processes (Halsey et al., 2018). Therefore, increases in environmental temperature led to an increase in metabolic rate, and consequently, to an increase in oxygen consumption, nutrient requirements, and water loss. This situation can have a limiting effect on energy balance and survival probability, especially in species living in hot and arid conditions (Clarke and Fraser, 2004; Angilletta, 2009).

However, some large-bodied reptile species can exhibit physiological characteristics that partially deviate from the classic definition of ectothermy. Organisms such as leather turtles (*Dermochelys coriacea*) and large crocodile species, in particular, can maintain their body temperatures relatively more stable against environmental fluctuations thanks to their high thermal inersias and, to a certain extent, metabolic and cardiovascular adjustments (Paladino et al., 1990; Seebacher et al., 1999). This situation is considered within the framework of the concepts of **“gigantothermy”** or limited regional endothermy, and it is suggested that body size slows down the rate of heat exchange, thus providing thermal stability (Bostrom et al., 2010).

However, these exceptional examples should be considered as specific cases where body size, circulatory system, and habitat characteristics converge, rather than representing the general physiological strategy of ectothermic vertebrates. In most amphibian and reptile species, metabolic rate remains highly sensitive to environmental temperature changes, and this is closely related to the species’ thermal tolerance limits, behavioral thermoregulation capacity, and energy budgets (Hochachka and Somero, 1984; Halsey, 2016).

In this context, the metabolism-temperature relationship stands out as a fundamental mechanism determining not only individual physiological performance but also growth rates, reproductive success, and population dynamics. If global temperature increases continue, the increase in metabolic demands seems inevitable, creating ecological and demographic costs for many amphibian and reptile species (Brown et al., 2004; Dillon et al., 2010).

## Water balance and osmoregulation

### Hydroregulation mechanisms

Water balance is one of the most fundamental physiological constraints for amphibians and reptiles living in terrestrial environments. These two taxonomic animal groups, being ectotherms and highly dependent on environmental humidity and temperature conditions, have developed distinctly different strategies for **hydroregulation**. In amphibians, in particular, water balance is closely linked to gas exchange and ion transport occurring directly through the skin; this makes them extremely susceptible to evaporative water loss (Hillman et al., 2008).

The thin and highly permeable skin structure of amphibians provides an advantage in terms of oxygen uptake and osmoregulation, but also leads to rapid water loss. In contrast, in reptiles, the keratinization of the epidermis and the presence of well-developed lipid barriers within the stratum corneum significantly limit cutaneous water loss, allowing this group to colonize drier habitats (Roberts and Lillywhite, 1980; Lillywhite, 2006).

These physiological differences lead to striking variations in cutaneous resistance values between species and habitats. According to reported values in the literature, cutaneous resistance in aquatic amphibians is extremely low, around 0.05 s/cm, while in reptiles adapted to desert ecosystems, this value can exceed 1000 s/cm. Comparatively, cutaneous resistance in mammals is generally reported in the range of 200–400 s/cm (Hillman et al., 2008). This contrast clearly explains why amphibians are tightly dependent on moist microhabitats and why reptiles can occupy a more hydrologically flexible ecological niche.

Terrestrial plethodontid salamanders represent one of the most extreme examples in terms of hydroregulation. This group is completely devoid of lungs and performs gas exchange entirely through the skin. Consequently, **cutaneous resistance** in these species is almost 0 s/cm, and water loss rates can be equivalent to the rate of free evaporation from an open water surface (Peterman and Semlitsch, 2014). This physiological necessity limits the distribution of plethodontid salamanders to high-humidity microhabitats, making them extremely vulnerable even to small-scale environmental changes (Feder, 1983).

Hydroregulation mechanisms involve not only the limitation of water loss but also ecological adaptations such as behavioral strategies, microhabitat selection, and activity timing (Shoemaker and Nagy, 1977; Hillman et al., 2008). However, habitat degradation and reduced microclimate heterogeneity severely restrict the effectiveness of these mechanisms, particularly in amphibians (Scheffers et al., 2014). Therefore, hydroregulation capacity stands out as a central concept in understanding the responses of amphibians and reptiles to climate change and habitat transformation (**Table 2**).

**Table 2:** Cutaneous water loss resistance by habitat type.

Group/species	Habitat	Resistance (s/cm)	Water loss (mg/cm²/h)	Reference
Amphibians				
Aquatic species	Permanent water	0.05-0.1	45-50	Hillman et al., 2008
Plethodontid salamanders	Humid forest	~0.0	~50	Peterman and Semlitsch, 2014
Semi-aquatic anurans	Wetlands	2.5-5.0	18-25	Withers et al., 1984
Terrestrial anurans	Forest floor	8-15	8-14	Young et al., 2005
Arboreal hylids	Tree canopy	45-68	1.8-4.0	Gomez et al., 2006
Reptiles				
Semi-aquatic turtles	Wetlands	90-180	0.8-2.0	Bentley, 2002
Temperate squamates	Terrestrial	150-250	0.5-1.5	Lillywhite, 2006
Desert reptiles	Arid	600-1200	0.08-0.25	Peterson, 1996
Mammals	Various	200-400	0.3-0.8	Hillman et al., 2008

Resistance values represent reported means or ranges from cited studies; sample sizes and confidence intervals vary across sources (n = 4–87 per group). Water loss rates (mg cm⁻² h⁻¹) are standardized to 25 °C where possible; significant thermal and humidity dependence of these values should be noted. Geographic bias toward North and South American, Australian, and European taxa is acknowledged. Values for ‘REPTILES’ include squamate-dominated datasets; chelonian and crocodilian data are substantially underrepresented. Inter-clade variation may be large and independent of habitat classification.

### Osmoregulation strategies

One of the most fundamental physiological requirements of terrestrial life is the effective maintenance of the ionic composition and water balance of the internal environment. Amphibians and reptiles have developed various osmoregulation strategies that enable them to adapt to different ecological environments. These strategies are carried out through the integration of multi-component processes such as kidney function, bladder utilization dynamics, ion transport mechanisms, and hormone-mediated physiological regulation (Shoemaker and Nagy, 1977).

In amphibians and reptiles, the kidneys function as the primary organs in water reabsorption and ion balance regulation. While kidney morphology is generally similar in these groups, significant species-specific adaptations are observed depending on the habitat and drought level (Dantzler, 1982). Amphibians generally have high filtration rates and limited urine concentration capacity, showing physiological dependence on humid environments. In contrast, in many reptile species, nitrogen excretion has shifted to the uric acid form, thus significantly reducing water loss (Bentley, 2002). This differentiation represents a critical evolutionary step in terrestrial adaptation.

The bladder serves as a critical physiological water reservoir for osmoregulation, especially in species living in arid habitats. In many amphibians and reptiles, the bladder is not only a storage structure but also an active regulatory organ where water is reabsorbed into the body when needed. For example, the desert tortoise *Gopherus agassizii* can survive by reabsorbing the fluid stored in its bladder during prolonged periods of drought. Although losses of up to 40% of body mass have been reported in this species during extremely dry years, individuals demonstrate physiological resilience, surviving even when total body water volume falls below 60% (Peterson, 1996). Similarly, in some amphibians, the bladder is not only a water reservoir but also an organ where ion recovery plays an active role (Shoemaker and Nagy, 1977).

The osmoregulation process does not rely solely on the passive properties of morphological and anatomical structures; It is also regulated by a strong endocrine control mechanism. Under dehydration or hyperosmotic stress, hormones such as aldosterone, arginine-vasotoxin (AVT), and angiotensin are known to rapidly become active. These hormones increase water and sodium reabsorption in the kidney, regulate vascular tone, and play a critical role in maintaining circulatory stability (McCormick and Bradshaw, 2006). Therefore, hormonal regulation acts as a fundamental homeostatic buffer, limiting the conversion of environmental stress into physiological effects.

These multifaceted osmoregulation mechanisms enable amphibians and reptiles to live in habitats with different hydrological regimes and maintain internal stability despite environmental drought, salinity changes, and fluctuations in moisture balance. However, increasing drought regimes, water regime disruptions, and decreased microhabitat moisture due to climate change are pushing the limits of these mechanisms, especially in species with high water dependence. Therefore, osmoregulation capacity is considered a key physiological marker in determining sensitivity to future environmental changes.

### Dehydration tolerance

One of the most critical physiological stressors for amphibians living in terrestrial environments is dehydration, which develops due to the loss of body water content. Anuran amphibians demonstrate remarkable resilience in this process, particularly in terms of maintaining their cardiovascular functions. Although plasma volume tends to decrease as dehydration progresses, the organism enables the maintenance of circulatory stability by providing reversible mobilization from interstitial fluid and intracellular water; this mechanism is considered crucial for survival (Hillman, 2018). Thus, perfusion and oxygen transport of critical tissues can be maintained even at very low hydration levels.

Recent studies have shown that dehydration is not merely a stressor in amphibians, but rather a synergistic threat that works in conjunction with temperature. Dupoué et al. (2020) demonstrated that under conditions where water loss and environmental temperature increase occur simultaneously, physiological performance deteriorates more rapidly, increasing vulnerability at the population level. These findings highlight that hydrological stress and thermal stress should not be considered independently, and that this interaction is a critical ecological pressure element, especially in drying habitats.

Experimental studies in the field of hydrothermal physiology show that water loss narrows thermal tolerance thresholds and that critical thermal limits are reduced to lower values. Riddell et al. (2016) showed that dehydration limits both physiological performance and behavioral thermoregulation capacity, which reduces the potential of species to respond to warming climate conditions, thus increasing vulnerability. This makes the fact that amphibians will be exposed to not only temperature increase but also decreasing ambient humidity and increased evaporation rates in future climate scenarios even more critical.

Dehydration tolerance varies significantly among species, and this variation is often related to microhabitat dependence, skin permeability, life history strategies, and ecological niche size. While species with strong dependence on humid microhabitats exhibit narrow tolerance ranges, broader physiological buffering mechanisms have been reported in species adapted to drier environments, allowing for the preservation of functional integrity despite water loss (Titon and Gomes, 2015). However, increasing desertification trends and decreasing microclimate heterogeneity can push the limits of tolerance even in species previously considered resilient. Therefore, dehydration tolerance should be considered not only as a trait determining physiological resilience at the individual level, but also as a key determinant of population continuity, geographical distribution limits, and vulnerability to climate change.

### Adaptive morphological features

Water loss reduction is achieved not only through behavioral strategies but also through specialized morphological adaptations. In amphibians, skin generally exhibits high permeability, resulting in significant physiological burden due to water loss; however, structural modifications that reduce water loss have emerged in some species.

In many anuran species, cutaneous resistance varies significantly depending on habitat type. Studies have shown that arboreal frogs have higher water loss resistance compared to sedentary terrestrial or aquatic species. For example, a comparative analysis of 25 species showed that many species with semi-permeable skin exhibited cutaneous resistance values close to 0, while arboreal hylid species showed high levels (e.g., ~63 s cm⁻¹), indicating a strong correlation between arboreal lifestyle and water loss resistance (Young et al., 2005).

One of the underlying mechanisms of this water loss resistance is the presence of lipid-rich, waterproof layers on the skin surface. Some members of the Hylidae and other arboreal frogs create a physiological barrier that limits water evaporation by secreting lipids from their skin and spreading them across their bodies. Studies on species such as *Pithecopus hypochondrialis* have reported that water loss rates following these lipid secretions are reduced by up to 96% compared to the free water surface; this demonstrates that the lipid coating is an effective evaporation-inhibiting mechanism (Gomez et al., 2006). Further evidence supports the findings that skin lipids minimize evaporative water loss. Experimental measurements on two arboreal hylid species showed that their water evaporation rates were significantly lower than those of their environmental replicates, and this effect was associated with the presence of the lipid layer (Amey and Grigg, 1995). Moreover, the relationship between water permeability and high cutaneous resistance is not restricted to arboreal species and has evolved independently in multiple ecological contexts. Selected groups of tree frogs from Western Australia, Africa, and South America also exhibit high cutaneous resistance and consequently, varying degrees of water conservation strategies; this variability reflects long-term selective pressures between water loss and microhabitat moisture (Withers et al., 1984).

These adaptive morphological features are not merely singular strategies for maintaining water balance, but evolutionary responses associated with ecological niches. Species living in drier or microclimatically variable environments, such as those with an arboreal lifestyle, have gained a selective advantage due to structural reasons that reduce water loss. Thus, physiological modifications such as lipid barriers and increases in skin resistance can be considered adaptations that increase the likelihood of survival under water stress. In addition to morphological adaptations, several amphibians exhibit behavioral strategies to reduce water loss. For example, some Australian tree frogs adopt a water-conserving posture that minimizes exposed surface area, thereby reducing evaporative loss (Withers et al., 1984). In contrast, arid-adapted burrowing frogs can retreat underground during dry periods and form a cocoon from shed skin layers, which acts as a waterproof barrier and allows them to survive prolonged droughts (Tracy et al., 2010).

### Renal adaptation and salt glands in reptiles

Reptiles have evolved diverse physiological mechanisms to regulate water and ion balance across desert, marine, and semi-aquatic environments. Central to these adaptations is the shift from ammonia- and urea-based nitrogen excretion (common in amphibians) to uric acid production, a metabolic adaptation that dramatically reduces obligatory water loss. Uric acid is sparingly soluble, allowing reptiles to excrete nitrogenous waste as a semi-solid paste with minimal water expenditure — a critical advantage in arid and marine environments (Dantzler, 1982; Minnich, 1982).

Many reptile taxa also possess specialized cephalic salt glands that supplement renal ion excretion under conditions of ionic stress. Marine iguanas (*Amblyrhynchus cristatus*) rely on nasal salt glands to excrete excess sodium and chloride ingested during marine algae foraging; without this extrarenal excretion pathway, plasma osmolality would rapidly reach lethal levels (Shoemaker and Nagy, 1977). Sea turtles possess large orbital lachrymal glands for concentrated salt excretion, enabling osmotic homeostasis in marine environments. In desert tortoises, water conservation is primarily achieved through behavioral dormancy combined with uric acid excretion — a strategy that contrasts fundamentally with the renal concentration mechanisms employed by desert-adapted birds and mammals (Minnich, 1982).

Despite these physiological advantages, climate-driven increases in drought frequency and rising ambient temperatures are beginning to challenge reptile osmoregulatory capacity. Studies on desert-adapted agamid lizards have shown that dehydration significantly impairs both locomotor performance and immune function even in drought-tolerant species, suggesting that physiological safety margins may be narrower than previously assumed under accelerating climate change conditions (Dupoué et al., 2020).

## Physiological responses to chemical pollutants

### Heavy metals and acidification

Amphibians are among the taxa directly exposed to environmental pollutants due to their high skin permeability; this amplifies the physiological effects of chemical stressors in aquatic and terrestrial habitats (Egea-Serrano et al., 2012). Heavy metals (e.g., aluminum, cadmium, copper, lead, zinc) accumulate in both aquatic and terrestrial habitats, leading to biochemical and physiological disturbances in amphibian and reptile populations (Freda, 1990).

Aluminum toxicity causes complex physiological effects in amphibians, particularly in relation to acidification processes. The toxic effect of aluminum depends not only on the environmental presence of the element but also on the pH, hardness, and dissolved organic carbon (DOC) content of the water (Freda, 1990). According to Freda’s (1990) review, aluminum toxicity can manifest in different ways in interaction with pH: under low pH conditions, aluminum solubility increases, enhancing the biologically harmful effects of the ionic Al³^+^; form, while at certain pH ranges, hydrogen ions modulate toxicity by preventing the binding of metal ions such as Cd²^+^; to the cell surface (Freda, 1990). Therefore, aluminum toxicity, when considered together with habitat acidification, reveals latent mechanisms of action.

The toxic effects of cadmium are significantly negative on growth, metamorphic development, and stress responses in amphibian larvae. For example, *in vitro* studies on the skin of *Pelophylax lessonae bergeri* have shown that cadmium exposure increases cellular stress responses such as metallothionein and heat shock proteins (HSPs) expression; this indicates that cadmium can directly cause molecular disruptions at the tissue level (Simoncelli et al., 2015). Although heavy metals such as cadmium bind to or stimulate cellular defense proteins, activating the organism’s protective potential, prolonged or high-dose exposure can result in metabolic stress, tissue damage, and lethal deterioration (Simoncelli et al., 2015).

The association of heavy metal pollution with stress responses has also been documented in reptiles. In fossorial (underground) reptiles exposed to high heavy metal concentrations in the soil, fecal glucocorticoid metabolite levels (e.g., corticosterone) are significantly increased; this indicates that heavy metals trigger physiological stress responses as a result of their endocrine-disrupting effects (e.g., field study on *Trogonophis wiegmanni*) (Martín et al., 2021). This finding reveals that heavy metal contamination not only causes tissue accumulation but also weakens the organism’s homeostatic capacity by affecting the regulation of stress hormones.

These heavy metal effects highlight the diversity of ecotoxicological mechanisms and their interactions with environmental parameters (pH, acidification, organic matter content). In particular, acidic conditions increase metal solubility, raising the level of exposure and toxicity potential of organisms; this indicates that the impact of environmental pollutants on amphibians and reptiles should be assessed not only in terms of concentration but also in conjunction with habitat chemistry.

### Pesticides and agricultural chemicals

The permeable skin structures of amphibians facilitate direct exposure to agricultural chemicals, significantly impacting the physiological and life history processes of these pollutants. Meta-analytic evaluations show that pesticides, fertilizers, and other agrochemicals lead to significant reductions in amphibian survival and body mass, and a serious increase in the frequency of abnormalities; overall, contamination has been found to have effects such as a 14% increase in mortality, approximately 7.5% reduced weight, and a 535% increased frequency of abnormalities (Egea-Serrano et al., 2012).

Pesticides and herbicides cause a wide range of effects, including developmental inhibition, behavioral disorders, immune suppression, endocrine system disruption, and morphological deformities. A number of laboratory and field studies have shown that pesticides delay larval development, prolonging the metamorphosis period, inhibiting growth, and often leading to deformities in appearance (Boone and James, 2003; Hayes et al., 2006).

Pesticide mixtures—particularly organophosphates, carbamates, and triazine class herbicides—have been shown to suppress the immune system even at low doses, reducing amphibian resistance to pathogens. This is associated with increased parasite load and disease susceptibility; atrazine exposure has been reported to increase trematode load and reduce immune cell count in *Lithobates pipiens* populations (Hayes et al., 2010).

The endocrine-disrupting effects of agricultural chemicals are closely related, particularly to pesticides that affect hormonal balance and developmental processes. Agrochemical-derived endocrine-disrupting compounds (EDCs) can affect both thyroid and gonadal hormone axes, leading to significant impairments in metamorphosis, sex differentiation, and reproductive capacity (e.g., observations of sex change and intersex phenotypes with herbicides such as atrazine) (Hayes et al., 2006; Mann et al., 2009; Adams et al., 2021).

Mann et al. A comprehensive review by (2009) demonstrated that agricultural chemicals not only have synergistic physiological effects but also interact with environmental factors to cause thyroid dysfunction and developmental delays. These findings highlight the complex effects of pesticides on endocrine homeostasis in amphibians and their potential implications for population sustainability (Mann et al., 2009).

Pesticide accumulation in aquatic and semi-aquatic habitats adjacent to agricultural areas, in addition to high mortality and sublethal effects, also brings with it additional ecological risks such as decreased reproductive success, behavioral changes (e.g., disruption of migration and mating behavior), and reduced population connectivity (e.g., effects of pesticide mixtures on population dynamics) (Adams et al., 2021).

These findings demonstrate that pesticides and agricultural chemicals are comprehensive stressors for amphibians, not only at the toxicological level but also at the ecological and life history levels. The effects of contamination vary depending on the type of chemical, life stage, duration of exposure, and environmental conditions, but the general trend is toward a wide range of physiological disturbances and population reductions; this poses a significant risk to sustainable biodiversity.

### Habitat change and pollution interactions

While habitat alteration and environmental pollutants are often considered separate stressors, current research indicates that these factors work together to create synergistic effects, leading to deeper physiological and population-level consequences for amphibians and reptiles. Large-scale anthropogenic activities, particularly mining, not only alter soil and water chemistry but also create comprehensive transformations in the structural characteristics of the habitat.

Studies have shown that forest cover loss, reduced coarse woody litter, and microclimatic disruption caused by mining activities can have stronger community-ecological impacts than the direct effects of metal pollution. A field study in Ontario showed that habitat structural variables such as canopy cover, coarse woody litter, and relative humidity had a higher explanatory power than metal concentration on amphibian and reptile species richness, abundance, and diversity; furthermore, decreasing canopy cover and increasing temperatures made metals more detrimental in terms of toxic effects (e.g., the combined effect of thermal stress and metal bioavailability). This finding suggests that synergistic interactions arising from the simultaneous presence of different environmental pressures can be more severe than the effects of individual factors (Sasaki et al., 2015).

These results suggest that habitat change creates a network of interactions that increase the toxicity of pollutants not only in the form of habitat loss or fragmentation, but also through the reshaping of microclimate conditions and ecosystem structure. For example, a decrease in canopy cover disrupts the water balance of amphibians by increasing exposure to solar radiation, ambient temperature, and evaporation rate, creating additional metabolic stress conditions that increase sensitivity to metals. When microclimate changes affect water potential and humidity regimes, tissue penetration and toxic effects of heavy metal ions also become more pronounced; this can have negative consequences on both population dynamics and individual physiological tolerances (e.g., the combined pressure of habitat desertification and pollutant effects) (Sasaki et al., 2015).

### Detoxification mechanisms and physiological responses

When amphibians and reptiles are exposed to environmental chemical stressors, a range of biochemical and morphological defense mechanisms are activated in their bodies. Pollutants, particularly heavy metals, increase oxidative stress by triggering the formation of reactive oxygen species (ROS) at the cellular level, and activate specialized detoxification systems to counteract them (Stark et al., 2022).

The liver plays a central role in the biotransformation of chemical toxins in amphibians and reptiles. Heavy metal exposure has been associated with significant changes in liver histology and increased detoxification capacity. For example, in anurans such as *Rhinella arenarum*, cadmium (Cd) exposure has been observed with liver histopathological changes such as ballooning in hepatocytes and hyperplasia of Kupffer cells; these findings suggest that the liver expands and increases its metabolic activity in response to metal toxins (Medına et al., 2016).

Metallothioneins (MTs) are low molecular weight proteins that bind toxins, particularly by complexing with Cd, Cu, Zn, and other metals, thereby limiting the toxic effects of their free ionic forms. These proteins are effective in binding metals and controlling their intracellular distribution, thus making a significant contribution to detoxification (Hogstrand and Haux, 1990).

The presence of MTs and their metal-binding capacity have also been directly demonstrated in amphibians. Studies have revealed the presence of MTs and their complexation with metals such as Cd, Cu, or Zn in species such as *Bombina orientalis*, *Bufo japonicus*, and *Hyla japonica*; this indicates that these proteins are active in limiting metal load at the cellular level (Suzuki and Kawamura, 1983).

In addition to metallothioneins, antioxidant enzymes and small molecule antioxidants also provide important defenses against metal toxicity. Glutathione (GSH) plays a significant role at the cellular level, both through its capacity to bind metal ions and its neutralization of reactive oxygen species. Increased enzymatic activities (e.g., superoxide dismutase — SOD, catalase — CAT, glutathione peroxidase — GPx, and glutathione S-transferase — GST) have been shown in antioxidant responses triggered by heavy metal exposure. For example, exposure to Zn, Cu, and Cd in *Lithobates catesbeianus* larvae led to a significant increase in the activity of antioxidant enzymes such as SOD, CAT, and GST in organs such as the liver and kidneys; this reflects a buffering role of the glutathione system against chemical stress (Carvalho et al., 2020).

Similarly, in amphibians such as *Pelophylax kl. esculentus*, increased metal content increased GSH levels in liver and skin tissues while altering some antioxidant enzyme activities. These types of responses are important biomarkers that monitor both tissue accumulation and antioxidant response as detectors of metal-induced oxidative stress (Prokić et al., 2016).

The effectiveness of these detoxification mechanisms is not limited to pollutant binding; it also depends on maintaining organ function. Histopathological changes in organs such as the liver, kidneys, and skin may indicate organ damage in cases of overload or disruption of detoxification systems. For example, in response to heavy metal exposure, the liver may exhibit adaptive changes such as growth and inflammation to enhance stress responses at the cellular level; however, prolonged or high-dose exposure can overwhelm these compensatory responses, leading to organ dysfunction (Medına et al., 2016).

In some reptile groups, such as subterranean amphisbaenians, a mechanism for the excretion of heavy metals through skin sloughing has also been identified; the observed relationship shows that reptiles in areas with higher soil metal levels have greater metal accumulation in their sloughed skin (Martín et al., 2021). This suggests that physiological processes such as skin shedding may provide an indirect pathway for metal excretion (Jones and Holladay, 2006).

These multi-layered detoxification systems form the basis for amphibians and reptiles to develop tolerance and resistance to environmental pollutants. However, these mechanisms have limited capacity: chronic exposures, high metal loads, and interaction with ecological stressors can deplete detoxification systems, leading to organ damage, metabolic disorders, and suppression of immune functions.

### Pesticides and endocrine disruption in reptiles

Reptiles have received comparatively less attention in ecotoxicological research, yet accumulating evidence indicates significant vulnerability to chemical pollutants through dietary bioaccumulation and direct exposure. Unlike amphibians, reptiles accumulate lipophilic contaminants such as organochlorine pesticides (OCPs), polychlorinated biphenyls (PCBs), and brominated flame retardants with high efficiency in fatty tissues; this biomagnification effect is particularly pronounced in top-predator species including alligators, large varanid lizards, and sea turtles (Guillette et al., 1994; Keller et al., 2004).

The endocrine-disrupting effects of environmental chemicals in reptiles are particularly consequential in species with temperature-dependent sex determination (TSD). Guillette et al. (1994) documented those juvenile alligators (*Alligator mississippiensis*) from a pesticide-contaminated lake in Florida exhibited reproductive abnormalities including masculinized and feminized gonadal morphology, accompanied by testosterone levels three to five times lower than reference populations. Because sex determination in these animals depends on incubation temperature rather than chromosomes, EDCs that mimic or antagonize steroid hormones represent a compounded threat when combined with already altered thermal regimes under climate change.

Sea turtles illustrate reptile ecotoxicology at the population scale. Bioaccumulation of OCPs, PCBs, and heavy metals in loggerhead (*Caretta caretta*) and green turtles (*Chelonia mydas*) is associated with immunosuppression, reduced reproductive output, and increased susceptibility to fibropapillomatosis — a disease involving both herpesvirus infection and pollutant-mediated immune dysfunction (Keller et al., 2004; Work et al., 2004). Because these migratory animals integrate pollutant loads across multiple ocean basins, contamination at distant foraging sites can manifest as physiological impairment in nesting populations thousands of kilometers away, making the full ecotoxicological burden of these species exceptionally difficult to assess.

Squamate reptiles in agricultural landscapes accumulate pesticide residues through prey consumption, with sublethal exposures documented to impair locomotor performance, reduce clutch size, and alter corticosterone stress responses. Studies of fence lizards (*Sceloporus undulatus*) near agricultural fields have found elevated hepatic enzyme activity consistent with detoxification stress (Hall and Henry, 1992). The intersection of pesticide exposure and thermal stress may be particularly consequential for reptiles, as elevated temperatures independently impair detoxification enzyme activity, potentially amplifying pesticide toxicity under warming conditions — a synergistic interaction that has received minimal systematic study.

## Disease responses and immune mechanisms

### Chytrid fungi (*Batrachochytrium dendrobatidis* and *B. salamandrivorans*)

*Batrachochytrium dendrobatidis* (*Bd*) and *Batrachochytrium salamandrivorans* (*Bsal*) are two chytrid fungal pathogens with widespread and devastating effects on amphibians. *Bd*, identified in 1998, has been associated with infections in at least 500 amphibian species worldwide, leading to massive population declines—causing local extinction events in many regions. The effects of *Bd* trigger chytridiomycosis, described by the IUCN and related scientific reviews as “the worst infectious disease recorded among vertebrates” (Berger et al., 1998; Daszak et al., 1999; Cunningham and Minting, 2008; Duffus and Cunningham, 2010).

*Bsal* is a more recently identified pathogen than *Bd* (2013), and is associated with high mortality rates, particularly in urodel amphibians (salamanders and newts). Dramatic declines, such as local population collapses of up to 99%, have been reported in European fire salamander (*Salamandra salamandra*) populations in the years following the identification of *Bsal*, indicating that *Bsal* poses a serious threat to salamander diversity (Spitzen-van der Sluijs et al., 2016).

Chytrid fungi specifically target the cutaneous (skin) surfaces of amphibians (Berger et al., 1998). Pathogens such as *Bd* and *Bsal* produce zoospores that infect the host’s skin, where the spores invade and multiply in epidermal cells, disrupting the skin’s most important physiological functions (Van Rooij et al., 2015). Since the skin structure of amphibians is critical for both osmoregulation and gas exchange, infection directly affects these vital processes (Voyles et al., 2019). In infected individuals: Electrolyte imbalance, ion transport in the skin is disrupted, which can affect heart rhythm and disrupt homeostasis (Voyles et al., 2019). Cardiac dysfunction, electrolyte imbalance and systemic effects create stress on the cardiovascular system (Voyles et al., 2019). Neurological effects, systemic stress and homeostatic disruption can affect nervous system function (Voyles et al., 2019). Immune system suppression, *Bd* produces factors that can weaken the host immune response; for example, *Bd* cells and toxins can inhibit lymphocyte proliferation and trigger apoptosis, which reduces the effectiveness of the immune response (Fites et al., 2013). The combination of these effects results in high mortality and population collapse, especially in sensitive species (Skerratt et al., 2007).

Although many amphibian species can be infected with *Bd*, the consequences of infection vary greatly from species to species. Some species exhibit resistant or tolerant behavior to the infection and carry the pathogen with low loads without developing serious disease; these species may play a carrier or reservoir role in enzootic environments (Cunningham and Minting, 2008; Duffus and Cunningham, 2010). For example, in common species such as the North American bullfrog (*Lithobates catesbeianus*), infection may occur but populations may recover or disease symptoms may be minimal, suggesting that these species contribute to the spread of *Bd*. For *Bsal*, the host range is narrower, and so far, serious effects have been shown, particularly in urodele amphibians. The mysteriously declining salamander populations in Europe following the identification of Bsal have highlighted the specific effects of this new pathogen on life cycle and immunological responses (Spitzen-van der Sluijs et al., 2016).

Amphibian immune responses to Bd involve both innate and adaptive mechanisms, including antimicrobial peptides, symbiotic skin microbiota, and lymphocyte-mediated responses. However, Bd can suppress lymphocyte proliferation and induce apoptosis, weakening host defenses and increasing susceptibility to infection (Fites et al., 2013; Colombo et al., 2015; Grogan et al., 2018).

### Ranaviruses

Ranaviruses (family Iridoviridae) are large, double-stranded DNA viruses that infect cold-blooded vertebrates (amphibians, reptiles, and fish). These viruses cause systemic infections in both wild populations and aquaculture settings, leading to significant mortality events (Cornell Wildlife Health Lab; ranavirus.org).

The most commonly reported ranavirus species in amphibians have been identified as Frog Virus 3 (FV3) and *Ambystoma tigrinum* virus (ATV). FV3 is the best-studied type of ranavirus and is associated with outbreaks and mass mortality in various anuran and urodel species. ATV, on the other hand, is particularly common in salamanders and has been associated with high mortality (WFV AG UTK via Gray et al., 2009; Rodrigues et al., 2024).

Ranavirus infections lead to systemic and severe pathology in amphibians. Clinical manifestations have been identified as follows hemorrhage and bleeding in the ventral skin, excessive lethargy, poor coordination, and abnormal swimming behavior, subcutaneous edema (swelling), necrosis in liver, spleen, and kidney tissues, and mass mortality events (death rates as high as 90%). These symptoms point to widespread tissue damage caused by ranaviruses on the kidney, liver, spleen, and hematopoietic tissues. Histopathological examinations show mass viral accumulation and necrosis, particularly in the renal proximal tubule epithelium, while edema and skin hemorrhages, along with systemic organ dysfunction, are observed (Mass.gov). Ranaviruses can be transmitted both through contaminated water in aquatic environments and through direct contact and ingestion of infected tissue; therefore, all life stages are potentially susceptible, and larval stages are particularly susceptible for most species (Cornell Wildlife Health Lab; ranavirus.org).

Host immune responses to ranavirus infections involve both innate and adaptive mechanisms, including interferon signaling, antibody production, and cytotoxic T-cell activity. However, immune competence varies across life stages, with larvae generally showing weaker antiviral responses and higher mortality rates. Viral gene products can also interfere with host immune signaling, contributing to persistence and increased pathogenicity (Grayfer et al., 2014; Tian et al., 2021; Grayfer et al., 2025).

### Co-infections and synergistic effects

Clinical and ecological studies indicate that the co-occurrence of pathogens such as *Batrachochytrium dendrobatidis* (*Bd*) and ranavirus within the same host can have substantial impacts on amphibian populations. Co-infections may lead to greater disease severity and potential synergistic effects compared with single-pathogen infections. Field surveys in Costa Rica, involving samples from 20 frog species, reported a prevalence of approximately 21.3% for Bd and 16.6% for ranavirus, and detected positive co-infection relationships in several species. These findings suggest that the presence of one pathogen can influence the transmission dynamics and infection status of the other, representing one of the first comprehensive field-scale demonstrations of such interactions (Whitfield et al., 2013; Erişmiş et al., 2019).

A comparable pattern has been observed in the Peruvian Andes. Studies in this region reported *Bd*–ranavirus co-infection rates of around 30% in both wild populations and collected individuals. These results demonstrate that the two pathogens can coexist within a single host and that each infection may affect the progression of the other (Warne et al., 2010). Research from the Himalayas and the tropical Andes further showed that co-infected individuals with high pathogen loads experienced more severe clinical outcomes, including increased mortality and elevated physiological stress, compared with uninfected or singly infected individuals (Warne et al., 2010).

Co-infections also involve interactions between pathogens. For instance, skin damage and immunosuppression caused by *Bd* may predispose hosts to ranavirus infection, while the systemic effects of ranavirus can facilitate the spread of *Bd* within the host. Because both pathogens target physical barriers and components of the immune system, their combined presence may result in synergistic pathogenic effects (Warne et al., 2010).

Both laboratory and field studies demonstrate that co-infections can have complex influences on infection intensity and population-level prevalence. Warne et al. (2010) reported similar Bd and ranavirus loads in wild and collected individuals, emphasizing not only the possibility of co-occurrence but also the potential for transmission. These findings indicate that amphibian disease dynamics cannot be fully explained by single-pathogen models; instead, multi-pathogen interactions play a more intricate role in shaping population trends, defense responses, and disease outcomes. To better understand the contribution of co-infections to global amphibian declines, broader studies encompassing different life stages, species, and ecological contexts are needed (**Table 3**).

**Table 3:** Major amphibian and reptile pathogens and their physiological impacts.

Pathogen	Type	Host range	Distribution	Mortality rate	Key impact	Reference
*Batrachochytrium dendrobatidis* (Bd)	Chytrid fungus	500+ species (all orders)	Global	30-90%	>200 species declining; 90+ extinct	Berger et al., 1998
*B. salamandrivorans* (Bsal)	Chytrid fungus	Urodeles	Europe, Asia	96-99%	99% decline in some populations	Spitzen-van der Sluijs et al., 2016
Frog Virus 3 (FV3)	Ranavirus	Anurans	Global	50-90%	Mass die-off events	Gray et al., 2009
*Ambystoma tigrinum* virus	Ranavirus	Urodeles	North America	70-95%	Larval stage mortality	Gray et al., 2009
Bd + Ranavirus co-infection	Mixed	Multiple species	Tropics	60-95%	Synergistic effects	Whitfield et al., 2013
*Ophidiomyces ophidiicola* (Snake Fungal Disease)	Ascomycete fungus	Serpentes (snakes)	North America, Europe	>50% in outbreaks	Ulcerative dermatitis; thermal stress exacerbates lesion progression	Lorch et al., 2015; Allender et al., 2015
Chelonian Alphaherpesvirus 5/Fibropapillomatosis	Alphaherpesvirus	Marine turtles (esp. *Chelonia mydas*)	Global (tropical/subtropical coasts)	Sublethal/chronic; population debilitation	Tumors, immunosuppression; linked to warming oceans and pollution	Work et al., 2004; Van Houtan et al., 2010
Ranavirus (chelonian strains)	Ranavirus (Iridoviridae)	Chelonia (esp. tortoises, box turtles); shared with amphibians	North America, Europe	Variable; hemorrhagic disease, organ failure	Cross-taxon transmission potential with amphibians; temperature-dependent immunity	Duffus et al., 2015

### Infectious diseases in reptiles: emerging pathogens and immune responses

While the preceding sections have focused primarily on amphibian pathogens, reptiles are also affected by a range of emerging infectious diseases that interact with environmental stressors and physiology in ecologically significant ways. Although the mechanistic literature on reptile immune physiology is less developed than for amphibians, accumulating evidence demonstrates that reptiles are susceptible to several devastating pathogens, some of which are exacerbated by climate change and habitat alteration.

Snake Fungal Disease (SFD), caused by the ascomycete fungus *Ophidiomyces ophidiicola* (syn. *Paranannizziopsis ophidiicola*), represents one of the most significant emerging infectious threats to wild snake populations in North America and Europe. The disease is characterized by cutaneous lesions, ulcerative dermatitis, abnormal molting, and in severe cases, systemic infection leading to death. Outbreak mortality rates in Eastern massasauga rattlesnakes (*Sistrurus catenatus*) and other pit vipers have been reported to exceed 50% in affected populations (Lorch et al., 2015). The pathophysiology of SFD involves direct invasion of the keratinized epidermis and underlying dermis by fungal hyphae, disrupting thermoregulatory behavior and immune competence. Critically, thermal stress appears to modulate disease progression: experimental studies have shown that snakes held at suboptimal temperatures exhibit more severe lesion progression, suggesting that climate-driven shifts in thermal regimes — particularly unseasonably cold or variable spring temperatures — may increase host vulnerability to *O. ophidiicola* (Allender et al., 2015). The immune response of snakes to SFD involves cutaneous inflammatory infiltration dominated by heterophils and macrophages; however, the capacity of the adaptive immune system to clear fungal infection remains poorly characterized compared to amphibian responses to chytrid fungi.

Chelonian Alphaherpesvirus 5 (ChHV5), associated with Fibropapillomatosis (FP) in marine turtles, represents another well-documented reptile disease with physio-ecological dimensions. FP is characterized by the development of benign fibropapillomatous tumors on the skin, eyes, and internal organs of green turtles (*Chelonia mydas*), and has been linked to coastal eutrophication, elevated sea surface temperatures, and exposure to marine pollutants including polycyclic aromatic hydrocarbons and heavy metals (Work et al., 2004; Van Houtan et al., 2010). The proposed mechanism involves pollutant-mediated immunosuppression that compromises the turtle’s ability to control ChHV5 replication. The prevalence of FP has increased markedly in warming coastal regions, with epizootics reported throughout the Indo-Pacific, Caribbean, and Mediterranean (Herbst, 1994). Immunological studies indicate that affected turtles exhibit reduced lymphocyte proliferative responses and altered heterophil-to-lymphocyte ratios, consistent with chronic immune suppression (Work et al., 2004). These findings illustrate the critical interaction between environmental stressors (pollution, warming), immune function, and viral disease progression in reptiles.

Ranaviruses also infect reptiles, and represent an important bridge between the disease ecologies of amphibians and reptiles. Several Ranavirus strains have been isolated from chelonians, including Emydid Herpesvirus 1-associated mass mortality events in gopher tortoises (*Gopherus polyphemus*) and box turtles (Terrapene spp.) in North America (Duffus et al., 2015). Clinical signs in reptile ranavirus infections include hemorrhagic necrosis of the oral mucosa, respiratory signs, and systemic organ failure. Because reptiles share aquatic habitats with amphibians in many ecosystems, ranaviruses may be transmitted across taxonomic boundaries, with amphibians potentially acting as reservoir hosts or amplifiers for strains that subsequently spill over into reptile populations (Duffus et al., 2015). Temperature-dependent immune suppression in reptiles — analogous to the HPI axis activation observed in amphibians — likely modulates the severity of ranavirus disease progression, though comparative immunological studies across herpetofauna remain scarce.

Comparative immunological perspectives further highlight important differences between amphibian and reptile responses to pathogens. Reptiles possess a more complex integument — keratinized scales and lipid-rich epidermis — that provides a more robust physical barrier against cutaneous pathogens compared to the highly permeable amphibian skin. However, reptiles rely heavily on temperature-dependent innate immune activity: phagocytic activity, complement activation, and natural killer cell function all decline significantly at temperatures below optimal ranges (Zapata et al., 1991). This thermal dependence of immunity creates a particular vulnerability for reptiles in habitats with increased thermal variability under climate change, where periods of suboptimal body temperature may coincide with peak pathogen exposure. In contrast to amphibians, the adaptive immune response of reptiles — particularly antibody-mediated immunity — is generally slower to mount, with immunoglobulin responses requiring days to weeks to develop (Zimmerman et al., 2014). These physiological constraints imply that reptile populations facing novel pathogens in rapidly changing environments may experience disproportionate mortality before adaptive immunity can provide protection, underscoring the conservation urgency of understanding reptile disease physiology in the context of global environmental change.

### Interactions between climate change and disease

Climate change is fundamentally reshaping not only individual physiological processes but also host-pathogen interactions. Changes in temperature and humidity conditions affect the growth, reproduction, and survival capacity of pathogens, as well as directly influencing the immune responses and stress physiology of host organisms. Therefore, the combined effect of environmental factors such as heat stress and water stress can create a network of ecotoxic interactions that increase disease susceptibility in amphibians.

Activation of the hypothalamus-pituitary-interrenal (HPI) axis in amphibians is a process linked to climate-induced stress. Heat stress and dehydration can trigger this axis, leading to elevated corticosteroid levels and suppressing both innate and lymphocyte-mediated immune responses via signaling pathways (Rollins-Smith and Woodhams, 2012). This mechanism can weaken the immune response to pathogens, increasing susceptibility, particularly to infections such as chytrid fungi (*Batrachochytrium dendrobatidis*) and ranaviruses.

The direct effects of climate change can also alter the phenology and spread of pathogens. For example, rising temperatures and milder winters can accelerate the growth and reproduction of pathogens such as Bd. This could lead to widespread chytridiomycosis outbreaks occurring more frequently during periods of high temperature or after winter; the theoretical basis for this is explained by the “thermal mismatch hypothesis”: when the different thermal tolerance curves of pathogens and hosts do not overlap, disease pressure increases when pathogens have a wider thermal tolerance than hosts. This hypothesis has been supported in laboratory and field studies, particularly in association with higher Bd infection prevalence at lower temperatures (Cohen et al., 2017; Rohr & Raffel, 2010; iucn-amphibian.org).

Indirect effects are also important. Changes in precipitation patterns and hydrology due to climate change can affect the transmission routes of pathogens. Since most amphibian pathogens are transmitted in aquatic environments (e.g., zoospores of the chytrid fungus), increased rainfall and waterlogging can increase the risk of infection, while dry periods can also affect pathogen survival in the water molecular environment. However, a decrease in liquid water may reduce the survival of moisture-dependent pathogens such as Bd; however, increased temperature and humidity variability can allow pathogens to exist in wider ecological niches than expected (e.g., detection of Bd in the Sonoran Desert) (Roth et al., 2023).

Furthermore, climate impacts on the immune system directly increase disease susceptibility. There is mechanistic evidence that heat and dehydration stress activate the HPI axis, leading to suppression of innate and adaptive immune responses; this suppression weakens defense mechanisms, particularly lymphocyte proliferation and skin antimicrobial peptide production. Hot and dry conditions have been shown to disrupt the microbiome community on the skin and reduce the number of beneficial microorganisms that compete with pathogens; This also shows that external environmental conditions further reduce the host’s resistance capacity (Rollins-Smith, 2017; Rollins-Smith and Le Sage, 2023).

All these interactions demonstrate that climate change is not only an environmental stressor but also a multidimensional process shaping host-immunity and pathogen dynamics. Rising temperatures, changing precipitation dynamics, and increasing extreme weather events associated with climate change can affect pathogen spread and host susceptibility in amphibians, creating more complex and increased disease risks than previously known.

## Climate change and adaptation capacity

### Phenological changes

Phenology refers to an organism’s timing of its seasonal cycles in response to environmental conditions, and global climate change is strongly affecting this timing in many species. Amphibians are particularly susceptible to phenological changes due to their high dependence on environmental temperature and precipitation regimes, and their tendency to exhibit phenological changes in response to climate change has been found to be 2–4 times stronger compared to other taxonomic groups (e.g., butterflies, birds, and plants) — a phenomenon supported by large-scale meta-analyses and observational studies (Parmesan, 2007; Walpole et al., 2012; Greenberg et al., 2014).

One of the best-documented examples of phenological changes is on reproductive migration and breeding timing. For example, the pygmy salamander (*Eurycea quadridigitata*) living in the southeastern United States delayed its migration time to breeding ponds by approximately 76 days compared to previous periods in a monitoring study spanning over 30 years (Todd et al., 2011), a striking example of the strong impact of climate change on phenological events.

Many studies have shown that early migration, breeding onset, and other seasonal activities in amphibians are associated with climate change. For example, in species such as *Bufo bufo* (common toad), early spring migrations were shown to start earlier, and this was associated with higher temperatures in long-term datasets; in the same study, some species exhibited a late breeding period or a delayed migration pattern, indicating heterogeneity in phenological responses among species (Todd et al., 2011).

In addition, a study on *Pelobates fuscus* in Italy showed that hot and dry spring conditions led to a delay in breeding migration, contrary to previous assumptions; This result highlights that not only temperature but also precipitation patterns play a critical role in phenological events (Dalpasso et al., 2023).

The ecological consequences of phenological changes are far-reaching. Changes in temperature and precipitation regimes can have direct effects on amphibian larval development, reproductive success, phenological mismatch with food sources, and tropical interactions; in particular, “false spring” events can increase the risk of early migration and reproduction in weather conditions unsuitable for reproduction, which can reduce the fitness of both individuals and populations (Buss et al., 2021).

A meta-analysis study shows that phenological responses of amphibians are mostly linked to temperature increase, but precipitation is also an important phenological trigger, especially in species with moisture-dependent reproductive cycles. This result supports complex patterns such as clinical phenological advancements as well as tidal and delayed responses (Ficetola and Maiorano, 2016).

Phenological changes have significant impacts not only on individual species but also on species associations and interactions at the ecosystem level. For example, changes in migration and breeding timing can affect predator-prey relationships, food cycling between larvae and adults, and competitive relationships; this can lead to disruption of broader community dynamics. Therefore, studying phenological changes in amphibians is a critical tool for understanding the impacts of climate change on life history strategies and population sustainability.

### Phenological shifts in reptiles

While amphibian-focused studies dominate phenological research, a growing body of evidence demonstrates that reptiles are also undergoing measurable shifts in emergence from dormancy, reproductive timing, and activity season length in response to climate warming (Schwanz and Janzen, 2008; Telemeco et al., 2009). In contrast to amphibians, reptile phenological responses are often more complex due to their reliance on behavioral thermoregulation and the direct thermal dependence of developmental processes including embryogenesis and sex determination.

Lizard species in temperate environments are showing earlier emergence from hibernation and longer active seasons (Kurnaz et al., 2016; Kurnaz and Şahin, 2024; Batum et al., 2025; Bayram et al., 2025). Long-term monitoring of European common lizards (*Zootoca vivipara*) has revealed that warming springs prompt earlier ovulation; however, these shifts may not be adaptive if prey availability does not correspondingly advance. Experimentally elevated temperatures in *Zootoca vivipara* led to earlier parturition but smaller offspring with reduced body condition, indicating a mismatch between thermal advancement and physiological readiness (Massot et al., 2008).

For oviparous reptiles, phenological shifts carry particularly significant consequences because nest-site thermal conditions directly determine incubation success and, in TSD species, offspring sex ratios. Earlier nesting in painted turtles (*Chrysemys picta*) correlates positively with spring warming in multi-decade datasets; however, warmer mid-summer incubation temperatures skew sex ratios strongly toward females, with projections suggesting near-complete feminization of some populations under high-emissions scenarios (Schwanz and Janzen, 2008). This creates demographic feedback unique to reptiles: phenological advancement does not prevent reproductive consequences of warming because incubation temperature — not adult activity timing — is the critical bottleneck.

Sea turtle nesting phenology is among the most thoroughly monitored reptile systems globally. Studies across Atlantic and Pacific nesting beaches document earlier mean nesting dates over multi-decade periods correlating with ocean surface temperature increases (Weishampel et al., 2004). These shifts interact with rising sand temperatures at nesting beaches, where temperatures above 33 °C impair embryo development and skew hatchling sex ratios. The convergence of phenological advancement and altered beach thermal regimes thus creates compounding physiological risks that cannot be fully mitigated through behavioral adjustment alone, underscoring that reptile phenological responses involve complex demographic trade-offs that deserve substantially greater research attention.

### Genetic adaptation and evolutionary potential

The accelerating pace of global climate change suggests that the current genetic makeup of many species may be insufficient to respond to this rapid change (Vaissi et al., 2023). In particular, in groups with long generation times and narrow environmental tolerances, such as amphibians and reptiles, whether existing genetic variation is sufficient for evolutionary adaptation is a significant source of uncertainty (Visser, 2008; Urban et al., 2013; Merilä and Hendry, 2013).

Genetic adaptation requires populations to exhibit persistent genetic changes driven by selection in response to environmental changes. However, direct, long-term evidence regarding genetic changes specific to climate change adaptation in amphibians and reptiles is still limited in the literature. A comprehensive meta-analysis noted that while many studies on these taxa documented phenotypic plasticity, very few studies directly measured time-dependent genetic adaptation (Urban et al., 2013). This raises questions about the potential role of genetic adaptation, as much of the available data is based on inferences derived from mechanistic and spatial variations (Urban et al., 2013).

Environmentally sensitive traits, such as temperature-dependent sex determination (TSD) in reptiles and some amphibians, interact with genetic mechanisms. For example, in many testudine and some lizard species, sex determination is entirely dependent on incubation temperature, a mechanism that can rapidly affect demographic sex ratios with climate change. Classical studies have revealed the demographic consequences of TSD in the context of climate change, but have suggested that these species may not be able to adapt rapidly genetically through selection; assessments have shown that the temperature effect “can inhibit male production at higher temperatures” and that this could lead to demographic collapses (Janzen, 1994). Specifically, Mitchell and Janzen (2010) modeled that the genetic variation of reptiles with TSD may not be sufficient to cope with high rates of climate change, highlighting the limits of their potential adaptive capacity (Cunningham et al., 2017).

These assessments highlight that the potential for genetic adaptation is limited not only by the size of the gene pool but also by the speed at which that gene pool responds to selection. While genetic variation in TSD susceptibility has been reported in reptiles and amphibians (e.g., laboratory studies have shown genetic variation in specific populations), whether this variation provides sufficient adaptive capacity remains an open question (Urban et al., 2013).

Sufficient genetic variation must exist within populations for genetic adaptation to be possible. Some studies have found evidence that phenotypic variation is genetically dependent, with different populations developing different thermal tolerances — for example, differences in heat tolerance and preference tendencies have been observed among Rana sylvatica populations, and these differences have been associated with genetic variation (Urban et al., 2013). However, direct evidence of such genetic differences as time-dependent adaptation (evolutionary change in the context of climate change) remains rare. Overall, meta-analyses show that both plasticity and genetic variation can shape the response of species to changing environmental conditions; however, it is a frequently repeated finding that plasticity is a more pronounced and widespread reactive mechanism in the short term. Therefore, a species’ capacity to adapt to climate change depends not only on genetic variation but also on whether the plasticity is adaptive or maladaptive (Urban et al., 2013).

Current scientific evidence suggests that the high level of genetic variation and rapid selection response required for genetic adaptation is likely limited in many amphibian and reptile populations. This suggests that short-term plasticity may play a more significant role than long-term genetic change under rapidly changing environmental conditions (Urban et al., 2013; Sinai et al., 2022). However, since current data are limited, direct measurement of genetic response through methods such as long-term genetic monitoring studies, collaborative environmental experiments, and genomic analyses is critical to addressing uncertainties in this area (Merilä and Hendry, 2013; Urban et al., 2013). Overall, current evidence suggests that while some genetic variation exists within populations, the rate of environmental change may exceed the pace of evolutionary adaptation in many amphibian and reptile species. Consequently, short-term phenotypic plasticity is likely to play a more immediate role than genetic adaptation in determining species persistence under rapid climate change (Merilä and Hendry, 2013; Urban et al., 2013; Fox et al., 2019).

### Distribution changes and dispersal restrictions

One of the most tangible biogeographic effects of global climate change is the reshaping of the geographical distribution boundaries of species (Kurnaz, 2022; Vaissi et al., 2025). The rapid shift in climate regimes causes many species to move from their area of suitable climatology to another geographic location; however, their capacity to reach these new areas is closely related to each species’ dispersal capabilities and the natural or anthropogenic barriers they encounter (Loarie et al., 2009; Schloss et al., 2012). Species with low dispersal capacity may not be able to respond to rapid changes in climate regimes by following their optimal distribution areas. This has been widely reported in both large-scale distribution models and field studies. Most reptiles and amphibians generally have limited individual mobility and a lack of corridors connecting suitable habitat fragments, making migration to new suitable areas difficult (Schloss et al., 2012; Urban et al., 2013).

Climate models predict that the geographic distribution of most species will shift northward or to higher altitudes. For example, a study covering 134 amphibian species in China predicted that a large proportion of the suitable climate zones for these species would shift northward and to higher altitudes; however, this shift is constrained for many species (e.g., Chinese amphibians) due to dispersal barriers and habitat fragmentation (Duan et al., 2016). Dispersal limits are not only restricted by the species’ own mobility capacity, but also by factors such as habitat fragmentation and land-use changes. Habitat loss and intensive human-use lands make it difficult for species to migrate to new suitable climate zones, increasing the risk of geographic untraceability; therefore, habitat connectivity and corridors are becoming increasingly important (Robillard et al., 2015). Many amphibian populations exhibit limited dispersal distances and strong site fidelity, which restricts their ability to track shifting climates (Smith and Green, 2005).

Dispersal limits are a critical threat, especially for species adapted to cold climates. Many cold-adapted amphibians in the Northern Hemisphere are shifting their suitable ranges northward due to the rapid pace of climate change, but their limited dispersal capacity prevents them from reaching these new areas, putting them at risk of distribution shrinkage and population extinction; this finding is replicated in both model-based predictions and field-focused research (Seaborn et al., 2025). These distribution shifts are associated with high extinction risks. A study by Thomas et al. (2003), a leading climate change model, demonstrated that perhaps the most fundamental consequence of environmental changes is the significant shrinking of existing species distributions and the isolation of populations; this isolation can trigger a loss of genetic diversity and a reduction in long-term adaptation potential (Urban et al., 2013).

When these factors are considered together, distribution changes and dispersal constraints are central to biogeography. When adapted to climate change, a “distribution bottleneck” can arise, such as (1) the emergence of new suitable areas, (2) the shrinking of existing habitats, and (3) the inability of species to reach these areas due to barriers. This bottleneck disrupts the population linkage of species, contributing to loss in microevolutionary processes and a high risk of extinction (Loarie et al., 2009; Schloss et al., 2012; Thomas et al., 2003; Urban et al., 2013).

### Microhabitat use and behavioral buffering

Microhabitats can reduce exposure to extreme heat, drought, or humidity-stressed conditions by providing critical climate buffer zones for ectothermic organisms such as amphibians and reptiles. Projections derived from large-scale macroclimate data often tend to overestimate the actual environmental conditions that species will encounter because these models do not account for micro-scale environmental heterogeneity (Scheffers et al., 2014). In this context, microhabitats can offer local microclimates that significantly reduce the thermal and hydric extremes to which organisms are exposed — microhabitat temperatures can be several °C lower than the ambient temperature and can reduce extreme temperature exposure durations by 10–30 times, which helps improve sensitivity models based on macroclimate predictions (e.g., forest shaded areas, epiphytes, under-shade microspaces) (Scheffers et al., 2014).

In the case of an amphibian, riparian microhabitats of habitat-specialized species like Geocrinia alba offer significantly lower dehydration risk and wetter, cooler microclimate conditions compared to the surrounding open areas. These microhabitats facilitate staying within the species’ physiological tolerance limits in terms of both water potential and temperature; in dry and hot conditions in open habitats, water loss increases rapidly, and physiological limits can be exceeded (e.g., Topt and CTmax values) (Hoffmann et al., 2020). This type of microhabitat utilization serves a critical buffering function for organisms during both intraday and seasonal extreme events.

The buffering capacity of microhabitats against climate pressures includes not only temperature but also moisture conditions. Factors such as soil moisture and shade cover in microhabitats maintain surface water balance, limiting water loss and reducing performance degradation due to dehydration. In this context, global analyses of altered microhabitats and microclimate refugia show that topographic heterogeneity reduces the risk of regional extinction of species (e.g., heat pressures for plant and insect populations in the UK are lower in the presence of microrefugia) (Suggitt et al., 2018).

Behavioral buffering represents another dimension of microhabitat utilization. Ectotherms can actively select more favorable microclimates by behaviorally choosing between thermal and hydric microspaces, which can mitigate the physiological effects of large-scale environmental stressors. For example, it has been experimentally shown that reptiles such as Uta stansburiana exhibit selective thermoregulation behaviors within complex microhabitat structures, which helps them maintain their body temperatures within their preferred ranges (Goller et al., 2014).

Therefore, microhabitat selection and behavioral buffering can be as important as species developing direct adaptive responses to macroclimate changes. The conservation and restoration of microhabitats should be considered an integral part of long-term climate change adaptation strategies; because these small-scale environmental heterogeneities affect the actual exposure and survival probabilities of species beyond existing macroclimate models, microclimate refugia and behavioral thermoregulation play a critical role in determining survival success, especially with the increasing extreme events of climate change (Kearney et al., 2009; Scheffers et al., 2014; Bernath-Plaisted et al., 2025). Burrowing behavior, including the use of soil cracks or animal burrows, represents an important microhabitat strategy that allows amphibians to avoid extreme thermal and hydric conditions (Schwarzkopf and Alford, 1996; Rothermel and Luhring, 2005; Pottier et al., 2025) (**Table 4**).

**Table 4:** Species vulnerability under climate change scenarios (†Reptile values in this table are extrapolated from amphibian-to-reptile vulnerability ratios in Cox et al., 2022 and Sinervo et al., 2024, not empirically derived; direct global reptile projections under named SSP scenarios are not yet available in the primary literature — see note below table).

Scenario	Temp. increase	Time	% Amphibians at risk	% Reptiles at risk	Primary threat	Reference
Optimistic						
SSP1-2.6	+1.5 °C	2050	~2%	*1-2%*	Tropical thermal stress	Pottier et al., 2025
Paris Target	+2.0 °C	2100	4-5%	*3-4%*	Upslope compression	von May et al., 2019
Moderate						
RCP 8.5	+2.5 °C	2070	~5-8%†	*~4-6%†*	Thermal + hydric stress	Sinervo et al., 2024
Current Trend	+3.0 °C	2100	4-25%*	*~3-15%†*	CTmax exceedance	von May et al., 2019
Pessimistic						
SSP5-8.5	+4.0 °C	2100	7.5%	*~5-10%†*	Physiological failure	Pottier et al., 2025
High Emissions	+4.5 °C	2100	~16%‡	*~10-15%‡*	Multi-stressor collapse	Urban, 2015

Values are derived as follows. (~2%) Current exposure: Pottier et al. (2025) report that 2% of 5,203 amphibian species are currently exposed to overheating events under shaded conditions. (4-5%) Paris Target/+2 °C: interpolated between the Pottier et al. (2025) baseline and von May et al. (2019), who project 4% CTmax exceedance at +3 °C for tropical Amazonian anurans. (*) Current Trend/+3 °C: von May et al. (2019) report that 4% of tropical lowland frogs will exceed CTmax and a further 25% will experience moderate physiological stress; the range 4–25% spans these two thresholds for tropical assemblages specifically. (7.5%) SSP5-8.5/+4 °C: Pottier et al. (2025) report this exact value globally for amphibians. (‡) High Emissions/+4.5 °C: Urban (2015) estimates ~16% of all species (not amphibian-specific) under RCP 8.5 (~4.3 °C); the value here refers to this all-taxa meta-analytic estimate and is flagged accordingly. (†) All reptile-specific percentages are extrapolated from amphibian-to-reptile vulnerability ratios in Cox et al. (2022) and Sinervo et al. (2024); Sinervo et al. (2024) additionally report that 38% of local populations among 30 desert reptile and amphibian species are projected to be lost within 50 years, providing a regional benchmark. Direct global reptile projections under named SSP scenarios are not yet available in the primary literature. These extrapolations are acknowledged limitations; readers should consult cited sources for full uncertainty ranges.

## Conclusion and future directions

This comprehensive review reveals that amphibians and reptiles face unprecedented environmental challenges that increasingly surpass their adaptive capabilities. Although these taxa exhibit physiological plasticity in thermal tolerance, osmoregulation, immune function, and detoxification, the accelerating pace of anthropogenic change threatens to overwhelm these compensatory mechanisms. Global models predict that a 4 °C warming scenario could drive substantial proportions of amphibian biodiversity beyond critical physiological thresholds, precipitating widespread extinctions (Pottier et al., 2025).

Three principal constraints emerge from current evidence. First, physiological boundaries are narrowing as thermal and hydric tolerance ranges fail to track rapidly shifting environmental baselines, leaving species vulnerable to extreme events and chronic stress (Pottier et al., 2025). Second, limited dispersal capacity prevents range adjustments necessary to track suitable climatic conditions, resulting in population isolation and elevated extinction risk (Sinervo et al., 2024). Third, multiple stressors interact synergistically rather than additively, with combinations of heat, dehydration, contaminants, pathogens, and habitat degradation generating disproportionate population-level impacts.

**Figure 1:**
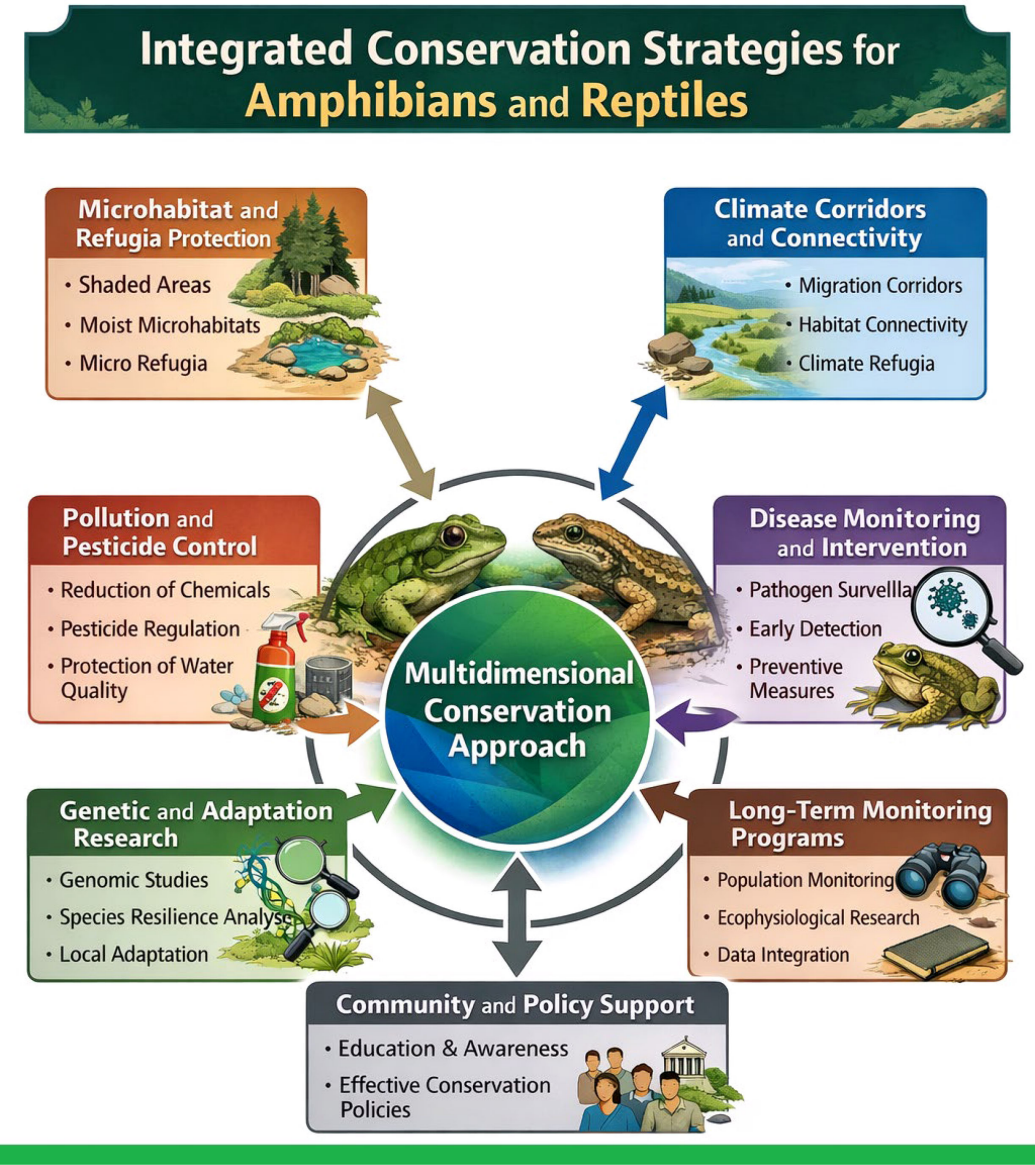
Schematic diagram illustrating integrated conservation strategies for amphibians and reptiles. The figure highlights a multidimensional conservation framework including microhabitat and refugia protection, climate corridors and connectivity, pollution and pesticide control, disease monitoring and intervention, genetic and adaptation research, long-term monitoring programs, and community and policy support. Together, these components aim to reduce environmental stress impacts, enhance species resilience, and promote long-term population sustainability.

Effective conservation requires integrated, multi-scale interventions. Habitat protection must extend beyond reserve establishment to encompass microhabitat heterogeneity and microclimatic refugia that buffer environmental extremes. Climate corridors facilitating latitudinal and elevational movement are essential where dispersal limitations constrain natural range shifts. Disease surveillance networks, pathogen load reduction strategies, and research into microbiota-mediated resistance are critical given the synergistic effects of environmental stress and infectious disease. Reducing chemical contaminant exposure through agricultural regulation and industrial oversight diminishes physiological burdens that compromise stress tolerance and immune competence. Long-term monitoring programs tracking population dynamics, phenological shifts, and disease interactions provide essential data for adaptive management, while genetic assessments inform strategies for maintaining evolutionary potential in fragmented populations (**Figure 1**).

Despite accumulating evidence of physiological responses to environmental change, fundamental knowledge gaps persist. The role of transgenerational plasticity and epigenetic inheritance in stress adaptation remains poorly characterized, as do the mechanistic bases of multiple stressor interactions, threshold dynamics, and irreversibility. Population-level variation in stress tolerance and local adaptation are inadequately documented, particularly for montane, insular, and peripheral populations where genetic differentiation may be pronounced. The molecular pathways linking environmental stress to immunosuppression and disease susceptibility require elucidation, as does the protective function of host-microbiome interactions. Fine-scale ecophysiological modeling incorporating microhabitat selection, behavioral thermoregulation, and microclimatic buffering is largely absent for most species. Genomic and systems biology approaches—including genome-wide scans for adaptation signatures, transcriptomic and metabolomic profiling, and multi-omics integration—remain underutilized despite their potential to illuminate mechanistic responses and identify vulnerable populations.

Addressing these challenges demands integrative research frameworks combining experimental ecophysiology, field ecology, genomics, and predictive modeling with sustained long-term monitoring. Conservation strategies must transition from static, habitat-centric models to dynamic, process-based frameworks that explicitly incorporate physiological constraints, disease dynamics, climate projections, and pollution impacts. Success will require not only scientific advances but also policy reform, stakeholder engagement, and public education. Given the physiological sensitivities and limited adaptive capacity documented across amphibian and reptile taxa, proactive, science-informed conservation action is essential to prevent catastrophic biodiversity loss in coming decades.
