# Supplementary material for: Physiological responses and adaptive mechanisms of amphibians and reptiles to multiple interacting environmental stressors: an integrative review
Source: Front Physiol. 2026 Apr 30;17:1785483. doi: 10.3389/fphys.2026.1785483 (PMC13171386; doi:10.3389/fphys.2026.1785483)
Supplement: Supplementary file 1 [file DataSheet1.pdf]

PRISMA-Style Literature Search Flow Diagram

Physiological Responses and Adaptive Mechanisms of Amphibians and Reptiles to Multiple Interacting Environmental Stressors: An Integrative Review

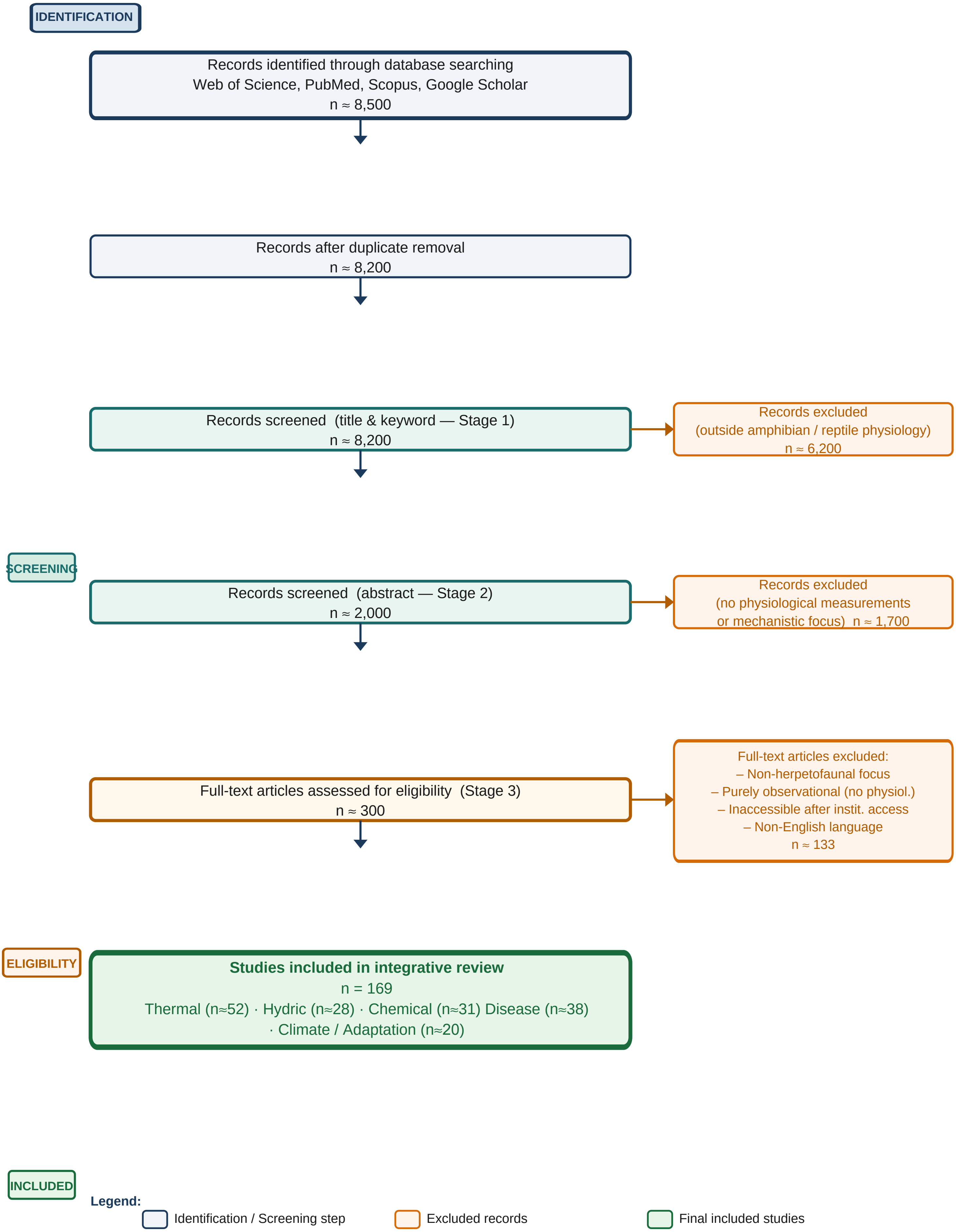

Screening followed general principles of transparent evidence synthesis (Liberati et al., 2009). Integrative review framework: Torraco (2005); Whittemore & Knafl (2005).
